# Proximal Humerus Epiphysiolysis as a Rare Cause of Fracture in Childbirth – A Case Report

**DOI:** 10.1055/s-0044-1779330

**Published:** 2024-12-27

**Authors:** Bárbara Noronha Teles, João Carlos Castro, Joana Ovídeo

**Affiliations:** 1 Ortopedia e Traumatologia, Hospital Professor Fernando Fonseca, Lisboa, Portugal; 2 Hospital Dona Estefânia, Centro Hospitalar Lisboa Central, Lisboa, Portugal

**Keywords:** epiphyses, slipped, fractures, bone, humeral fractures, infant, newborn

## Abstract

Proximal humeral epiphysiolysis (PHE) are rare at 10.1/100,000 births and there are few cases described in the literature. We present the case of a newborn diagnosed with PHE submitted to conservative treatment. In six weeks he had complete mobility and extensive bone callus. As a very rare situation, rapid diagnosis is imperative, for which ultrasound is decisive and the attitude must be conservative and expectant, given a very rapid and expected evolution towards consolidation for normal function. This case reinforces the previous knowledge that these lesions typically evolve favorably, and post-traumatic sequelae are not expected.

## Introduction

Proximal humeral epiphysiolysis at birth are rare at 10.1/100,000 births 1 and typically occurs after a traumatic birth. There are a few cases described in the literature. 2 Diagnosis is challenging as X-rays can be inconclusive. Ultrasound is a simple, readily available, and inexpensive modality for the diagnosis of birth-related fractures of the humerus. 2 The treatment is usually non-surgical. 3

## Clinical Case

**Fig. 1:**
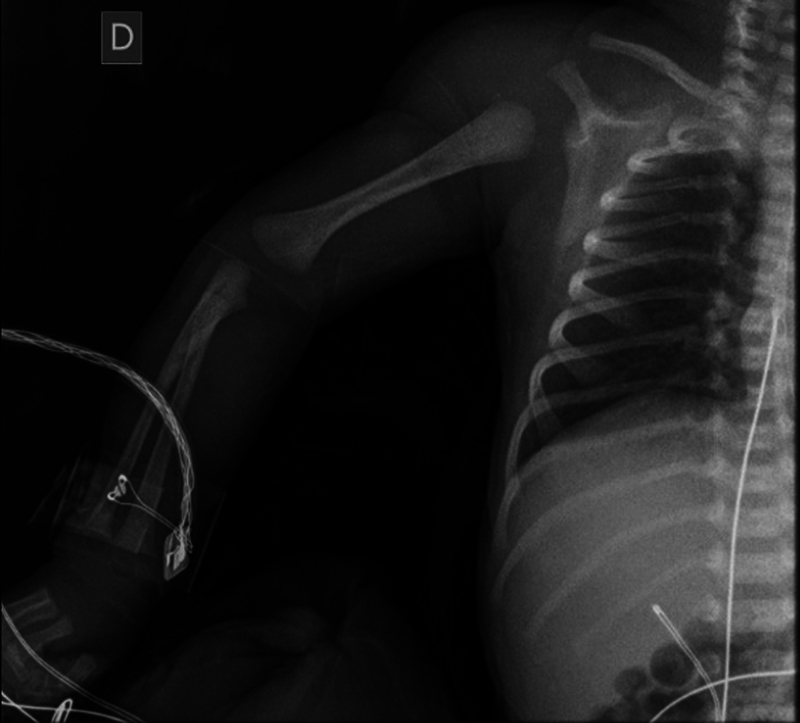
Newborn X-ray.

**Fig. 2:**
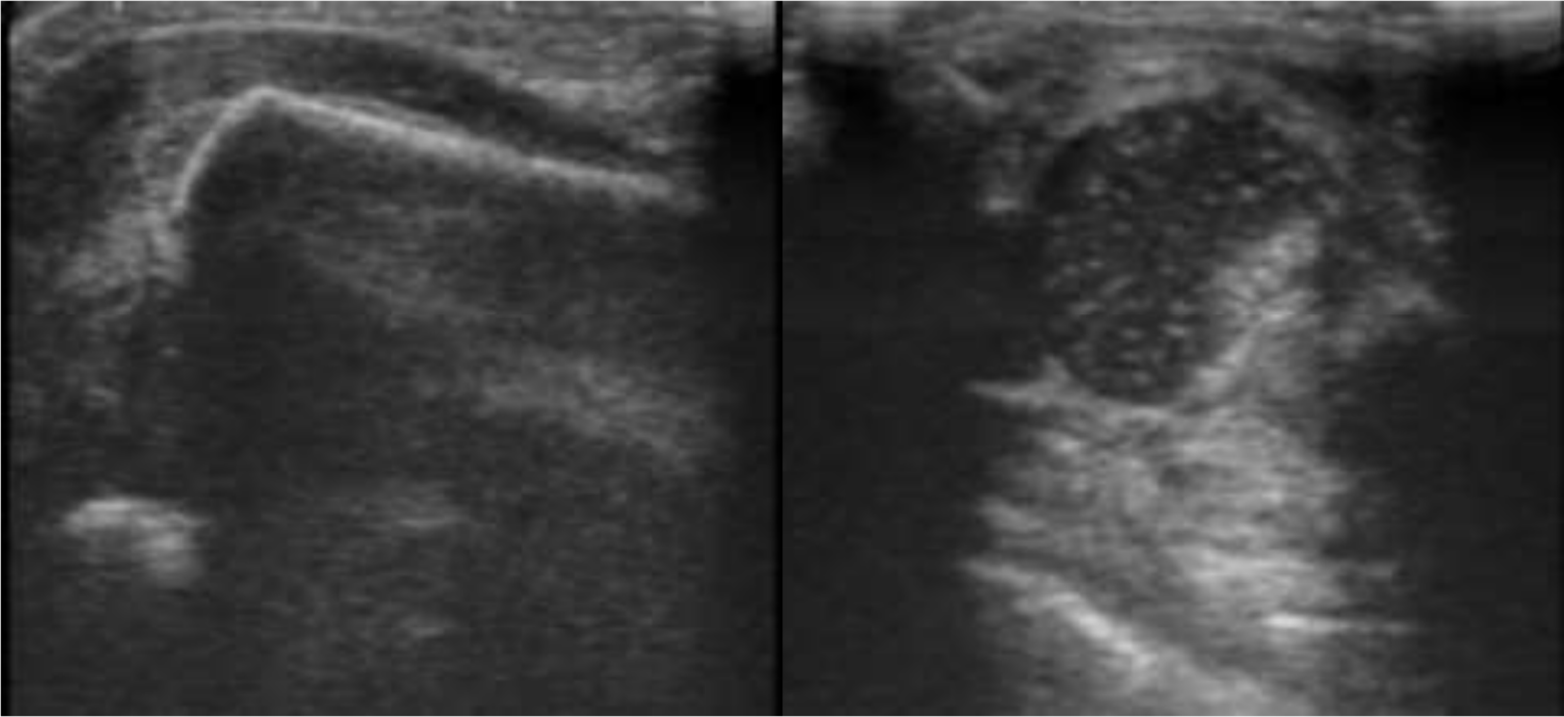
Newborn shoulder ultrasonography.

We present the case of a newborn, twin pregnancy, born at 35 weeks of dystocic delivery with breech presentation of a primiparous mother, with birth weight 2,600 kg, with mobility and asymmetrical Moro reflex on the right, increased volume and diffuse ecchymosis in the ipsilateral shoulder. He performed radiography ( Fig. 1 ) and later the ultrasound ( Fig. 2 ) confirmed posterior deviation of the humeral epiphysis in relation to the diaphyseal axis of the humerus, a finding compatible with fracture injury with epiphysiolysis. Since it was a traumatic birth other musculoskeletal injuries were excluded, as well as such as brachial plexus injury. Since he was delivered by breech presentation, despite a normal hip physical exam and the absence of family history, at six weeks was submitted to hip ultrasound, that was normal (Graf classification I).

According to the literature, a conservative treatment was chosen. The right arm was bandaged to the chest in the neutral position for two weeks.

**Fig. 3:**
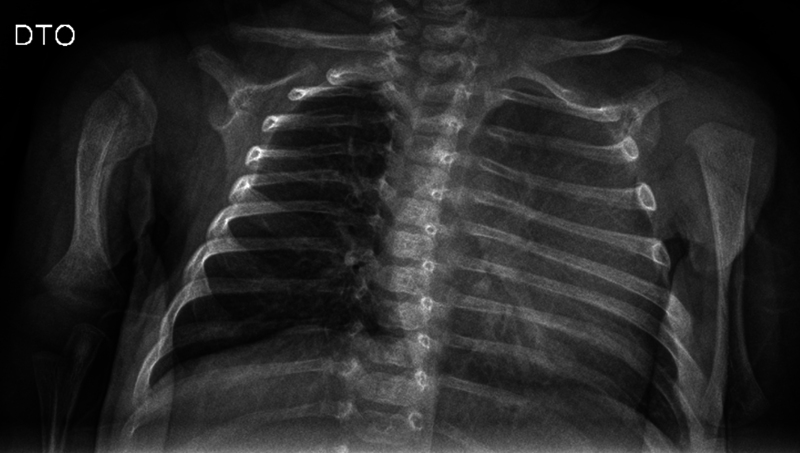
X-ray at 6 weeks.

**Fig. 4:**
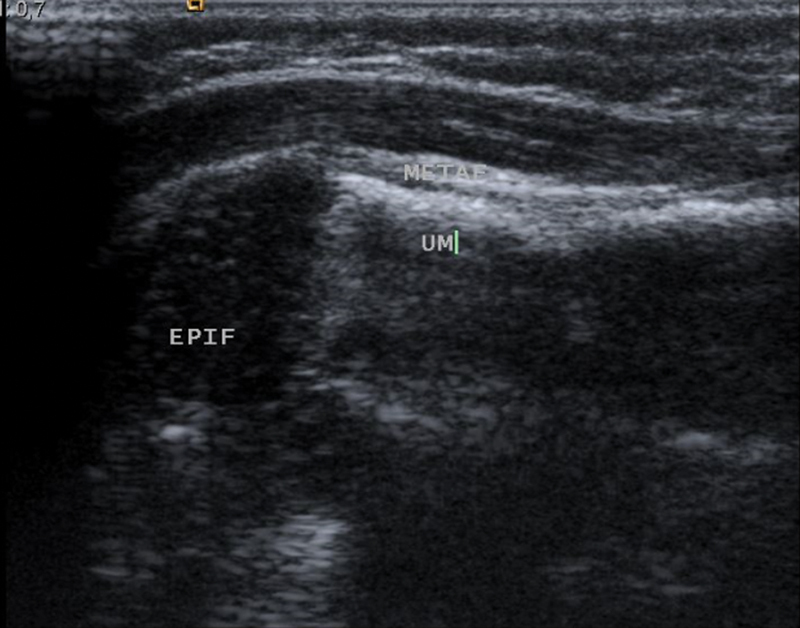
6-week ultrasonography.

**Fig. 5:**
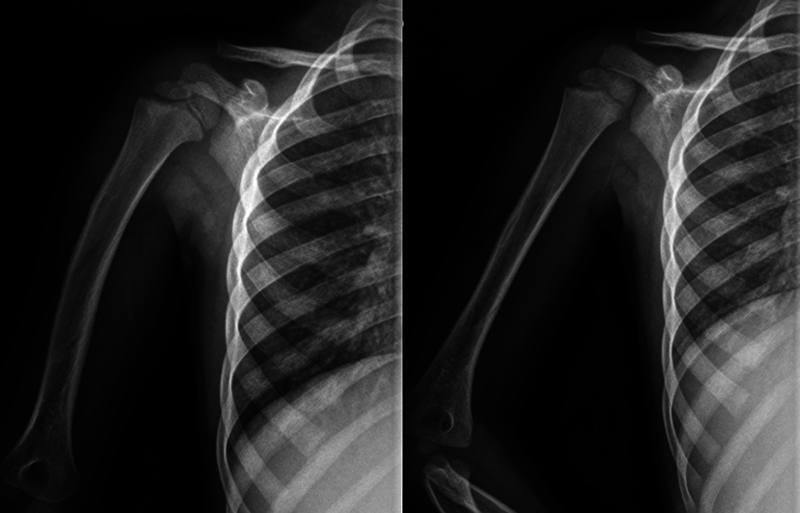
X-ray at 4 years old.

The authors performed a serial clinical and imaging follow-up. At four weeks, he spontaneously mobilized the right upper limb, without apparent pain, and at six weeks he had complete mobility and extensive bone callus on x-ray ( Fig. 3 ) and ultrasonography ( Fig. 4 ). With one year of evolution, the clinical examination were normal and an almost complete bone remodeling with open physis was observed. At four years of age, he present with full range of motion, symmetrical strength, no residual complaints. Radiologically remodeled without any rotacional deformity ( Fig. 5 ).

It was brought to the attention of the patient's guardian whether the data concerning the case could be submitted for publication, and she consented by signing the informed consent form.

## Discussion

A fracture that occurs in the first week of life with no known postnatal trauma is considered a birth fracture. 4 During the descent down the birth canal, the infant's arm can be placed in a variety of compromised positions, that can result in a physeal fracture of the proximal humerus, normally corresponding to extension lesions. 5 However, fractures of the clavicle are much more common during delivery than are fractures of the proximal humerus. Vaginal deliveries, breech presentation, prolonged labor from primiparous mothers, and macrosomia (>4.5 kg) are risk factors for a birth fracture. Birth fractures of the proximal humerus are classic physeal separations or Salter-Harris type I injuries. Reports of Salter-Harris type II fractures are rare but are likely underreported because, in many infants, the proximal humerus is not yet ossified. 6

The proximal physis of the humerus contributes 80% of the longitudinal growth of that bone, so fractures at that site exhibit considerable remodeling potential. The configuration of the epiphyseal plate and the thickness of the periosteum surrounding the epiphysis make slight to moderate displacements relatively stable injuries. 5

Regarding the diagnosis, ultrasonography is an accessible and inexpensive imaging modality for the diagnosis of proximal humerus fractures in neonates. Advantages of ultrasound are it may show greater details of the deformity compared to x-ray without exposure to radiation. 7 The sensitivity of ultrasound is 94% and the specificity 100% for diagnosis of proximal humerus fractures in children. 8

In neonates, the treatment is almost always nonoperative due to the immense remodeling power of the growth plate. Treatment with gentle swaddling is effective in this age group without long-term deformity. 8 9

Previous reviews in the literature of cases of proximal humerus epiphysiolysis in newborns demonstrated fracture union an average within three weeks, and radiographs at the age of six months demonstrated remodeling of the fracture 2 with conservative treatment.

As a very rare situation, rapid diagnosis is imperative, for which ultrasound is decisive and the attitude must be conservative and expectant, given a very rapid and expected evolution towards consolidation and normal function. This case reinforces the previous knowledge that these lesions typically evolve favorably, and post-traumatic sequelae are not expected.
