# Supplementary material for: Proximal Humerus Epiphysiolysis as a Rare Cause of Fracture in Childbirth – A Case Report
Source: Rev Bras Ortop (Sao Paulo). 2024 Dec 27;59(Suppl 2):e243–6. doi: 10.1055/s-0044-1779330 (PMC11679611; doi:10.1055/s-0044-1779330)
Supplement: Related texts

# Research article: Epifisiólise proximal do úmero como causa rara de fratura no parto – Relato de caso

**Keywords:** fraturas ósseas, fraturas do úmero, epifise deslocada, recém-nascido

## Abstract

A epifisiólise proximal do úmero (EPU) é rara, presente em 10,1/100.000 nascimentos e há poucos casos descritos na literatura. Apresentamos o caso de um recém-nascido com diagnóstico de EPU submetido a tratamento conservador. Em seis semanas apresentava mobilidade completa e extenso calo ósseo. Sendo uma situação muito rara, é imperativo um diagnóstico rápido, para o qual a ecografia é decisiva e a abordagem deve ser conservadora e expectante, dada uma evolução muito rápida e esperada no sentido da consolidação para a função normal. Este caso reforça o conhecimento prévio de que essas lesões normalmente evoluem favoravelmente e não são esperadas sequelas pós-traumáticas.

## Introdução

A epifisólise proximal do úmero no nascimento é rara, ocorrendo em 10,1 a cada 100.000 nascimentos 1 e geralmente ocorre após um parto traumático. Há poucos casos descritos na literatura. 2 O diagnóstico é desafiador, pois as radiografias podem ser inconclusivas. A ultrassonografia é uma modalidade simples, prontamente disponível e de baixo custo para o diagnóstico de fraturas do úmero relacionadas ao nascimento. 2 O tratamento geralmente é não cirúrgico. 3

## Caso clínico

Apresentamos o caso de um recém-nascido, fruto de uma gestação gemelar, que nasceu às 35 semanas de um parto distócico com apresentação pélvica em uma mãe primípara. O peso ao nascer foi de 2.600 kg. O bebê apresentou mobilidade e reflexo de Moro assimétrico à direita, juntamente com aumento de volume e equimoses difusas no ombro ipsilateral. O recém-nascido foi submetido a uma radiografia ( Fig. 1 ) e posteriormente a ultrassonografia ( Fig. 2 ) confirmou desvio posterior da epífise umeral em relação ao eixo diafisário do úmero, achado compatível com lesão por fratura com epifisiólise. Por se tratar de um parto traumático, foram excluídas outras lesões musculoesqueléticas, bem como a lesão do plexo braquial. Como nasceu em apresentação pélvica, apesar do exame físico da quadril normal e da ausência de antecedentes familiares, às seis semanas foi submetido a ecografia da quadril, que foi normal (classificação Graf I).

**Fig. 1:** Radiografia do recém-nascido.

**Fig. 2:** Ultrassonografia do ombro do recém-nascido.

De acordo com a literatura, optou-se por tratamento conservador. O braço direito foi enfaixado até o peito em posição neutra por duas semanas.

Os autores realizaram acompanhamento clínico e de imagem seriado. Às quatro semanas mobilizou espontaneamente o membro superior direito, sem dor aparente, e às seis semanas apresentava mobilidade completa e extenso calo ósseo na radiografia ( Fig. 3 ) e ultrassonografia ( Fig. 4 ). Com um ano de evolução, o exame clínico era normal e observou-se remodelação óssea quase completa com fise aberta. Aos quatro anos apresenta amplitude de movimento plena, força simétrica, sem queixas residuais. Remodelado radiologicamente sem qualquer deformidade rotacional ( Fig. 5 ).

**Fig. 3:** Radiografia com seis semanas.

**Fig. 4:** Ultrassonografia com seis semanas.

**Fig. 5:** Radiografia aos quatro anos.

Foi levado ao conhecimento da responsável pelo paciente se os dados referentes ao caso poderiam ser submetidos para publicação, e ela consentiu assinando o Termo de Consentimento Livre e Esclarecido.

## Discussão

Uma fratura que ocorre na primeira semana de vida sem trauma pós-natal conhecido é considerada uma fratura de nascimento. 4 Durante a descida pelo canal de parto, o braço do bebê pode ser colocado em uma variedade de posições comprometidas, o que pode resultar em uma fratura da fise do úmero proximal, geralmente correspondendo a lesões por extensão. 5 No entanto, as fraturas da clavícula são muito mais comuns durante o parto do que as fraturas do úmero proximal. Partos vaginais, apresentação pélvica, trabalho de parto prolongado de mães primíparas e macrossomia (>4,5 kg) são fatores de risco para fratura de nascimento. As fraturas de nascimento do úmero proximal são separações fisárias clássicas ou lesões Salter-Harris tipo I. Relatos de fraturas Salter-Harris tipo II são raros, mas provavelmente são subnotificados porque, em muitos bebês, o úmero proximal ainda não está ossificado. 6

A fise proximal do úmero contribui com 80% do crescimento longitudinal desse osso, portanto as fraturas nesse local apresentam considerável potencial de remodelação. A configuração da placa epifisária e a espessura do periósteo que circunda a epífise tornam lesões com deslocamentos leves a moderados relativamente estáveis. 5

Quanto ao diagnóstico, a ultrassonografia é uma modalidade de imagem acessível e barata para o diagnóstico de fraturas do úmero proximal em neonatos. As vantagens do ultrassom são que ele pode mostrar maiores detalhes da deformidade em comparação ao raio X sem exposição à radiação. 7 A sensibilidade da ultrassonografia é de 94% e a especificidade de 100% para diagnóstico de fraturas proximais do úmero em crianças. 8

Em recém-nascidos, o tratamento é quase sempre não cirúrgico devido ao imenso poder de remodelação da placa de crescimento. O tratamento com envolvimento cuidadoso é eficaz nesse grupo etário, sem resultar em deformidades a longo prazo. 8 9

Revisões anteriores na literatura de casos de epifisiólise proximal do úmero em recém-nascidos demonstraram consolidação da fratura em média dentro de três semanas, e radiografias aos seis meses de idade demonstraram remodelamento da fratura 2 com tratamento conservador.

Sendo uma situação muito rara, é imperativo um diagnóstico rápido, para o qual a ecografia é decisiva e a atitude deve ser conservadora e expectante, dada uma evolução muito rápida e esperada para a consolidação e função normal. Este caso reforça o conhecimento prévio de que essas lesões normalmente evoluem favoravelmente e não são esperadas sequelas pós-traumáticas.
